# Correction: Leaving no one behind on the road to Universal Health Coverage: The Kerala story

**DOI:** 10.1186/s12939-024-02195-3

**Published:** 2024-07-09

**Authors:** International Journal for Equity in Health

**Affiliations:** London, UK

**Correction: Int J Equity Health 22, 165 (2023); 22, 193 (2023); 22, 176 (2023); 22, 197 (2023); 23, 14 (2024)**

**https://doi.org/10.1186/s12939-023-01978-4**

**https://doi.org/10.1186/s12939-023-01981-9**

**https://doi.org/10.1186/s12939-023-01991-7**

**https://doi.org/10.1186/s12939-023-02005-2**

**https://doi.org/10.1186/s12939-023-02090-3**

The original publications [1–5] did not contain an abstract in Malayalam. To increase the accessibility of these publications the Malayalam language abstracts of these articles are available as Supplementary files.

10.1186/s12939-023-01978-4

Understanding Dalit equity: a critical analysis of primary health care policy discourse of Kerala in the context of ‘Aardram’ mission

Sreenidhi Sreekumar

10.1186/s12939-023-01981-9

Equity issues in gender-affirming medical care in Kerala: a reflective commentary

K. Rajasekharan Nayar & S. Vinu

10.1186/s12939-023-01991-7

Factors affecting ability of TB patients to follow treatment guidelines “ applying a capability approach”

B Aravind Chandru and Ravi Prasad Varma

10.1186/s12939-023-02005-2

Assessing inequalities in publicly funded health insurance scheme coverage and out-of-pocket expenditure for hospitalization: findings from a household survey in Kerala

Santosh Kumar Sharma, Jaison Joseph, Hari Sankar D, Devaki Nambiar

10.1186/s12939-023-02090-3

What our children lost and gained at the time of school closure during the Covid-19 pandemic: a study on psychological distress, behavioural concerns and protective factors of resilience among preschool children in Kerala, India.

Jose Vincent, Resmi Madhusoodanan Santhakumari, Anjana Nalinakumari Kesavan Nair, Anisha Sharahudeen, Asvini K.P, Meenu Maheswari Suresh, Mathew J. Valamparampil, Gayathri A.V, Chintha Sujatha and Anish Thekkumkara Surendran

### Supplementary Information

Supplementary Material 1.

## References

[1] Sreekumar S (2023). Understanding Dalit equity: a critical analysis of primary health care policy discourse of Kerala in the context of ‘Aardram’ mission. Int J Equity Health.

[2] Nayar KR, Vinu S (2023). Equity issues in gender-affirming medical care in Kerala: a reflective commentary. Int J Equity Health.

[3] Chandru BA, Varma RP (2023). Factors affecting ability of TB patients to follow treatment guidelines – applying a capability approach. Int J Equity Health.

[4] Sharma SK, Joseph J, Hari Sankar D. et al. Assessing inequalities in publicly funded health insurance scheme coverage and out-of-pocket expenditure for hospitalization: findings from a household survey in Kerala. Int J Equity Health. 2023;22:197. 10.1186/s12939-023-02005-2. 10.1186/s12939-023-02005-2 PMC10537906 37759247

[5] Vincent J, Santhakumari RM, Nalinakumari Kesavan Nair A. et al. What our children lost and gained at the time of school closure during the Covid-19 pandemic: a study on psychological distress, behavioural concerns and protective factors of resilience among preschool children in Kerala, India. Int J Equity Health. 2024;23:14. 10.1186/s12939-023-02090-3. 10.1186/s12939-023-02090-3 PMC10807164 38263155

