# Supplementary material for: Correction: Leaving no one behind on the road to Universal Health Coverage: The Kerala story
Source: Int J Equity Health. 2024 Jul 9;23:137. doi: 10.1186/s12939-024-02195-3 (PMC11232204; doi:10.1186/s12939-024-02195-3)
Supplement: Supplementary file 1 — Supplementary Material 1. [file 12939_2024_2195_MOESM1_ESM.zip › 12939-2023-1978-4 Sreekumar.pdf]

Understanding Dalit equity: a critical analysis of primary health care policy discourse of Kerala in the context of 'Aardram' mission

Sreenidhi Sreekumar

ദളിതരും തുല്യനീതിയും: ആർദ്രം ആരോഗ്യ ദൗത്യത്തിന്റെ പശ്ചാത്തലത്തിൽ കേരളത്തിലെ പ്രാഥമിക ആരോഗ്യ നയങ്ങളുടെയും ചിന്തകളുടെയും ഒരു വിമർശനാത്മക പരിശോധന

ശ്രീനിധി ശ്രീകുമാർ

ആമുഖം: സംസ്ഥാനത്തെ സാമൂഹ്യാരോഗ്യരംഗത്തിന്റെ വീണ്ടെടുപ്പും ശാക്തീകരണവും ലക്ഷ്യംവെച്ച് 2017ൽ കേരള സർക്കാർ തുടക്കംകുറിച്ച പദ്ധതിയാണ് ആർദ്രം മിഷൻ. കുടുംബാരോഗ്യ കേന്ദ്രങ്ങൾ വഴി സമഗ്രമായ പ്രാഥമികാരോഗ്യപരിരക്ഷ ലഭ്യമാക്കുക എന്നതായിരുന്നു ഇതിന്റെ പ്രധാന നയപരിപാടി. ഇതിലൂടെ ആരോഗ്യ രംഗത്ത് സാമൂഹിക നീതി ഉറപ്പാക്കുക എന്നതുമായിരിന്നു ആർദ്രത്തിന്റെ പ്രധാന ലക്ഷ്യങ്ങൾ. ഈ പശ്ചാത്തലത്തിൽ, ആരോഗ്യമേഖലയിൽ ജാതീയമായി സംഭവിക്കാവുന്ന അനീതികളെ കുടുംബാരോഗ്യ നയം എങ്ങനെ കൈകാര്യം ചെയ്യുന്നു എന്ന് മനസിലാക്കുവാനാണ് ഈ പഠനം ഊന്നൽ നൽകിയത്.

രീതിശാസ്ത്രം: ഈ പഠനത്തിനായി ക്രിട്ടിക്കൽ ഡിസ്കോർസ് അനാലിസിസ് എന്ന രീതിയാണ് അവലംബിച്ചത്. ഇതിനായി നിലവിൽ ലഭ്യമായിട്ടുള്ള കുടുംബാരോഗ്യ നയത്തിന്റെ രേഖകളും, ആരോഗ്യവകുപ്പിലെ മുതിർന്ന ഉദ്യോഗസ്ഥരുമായി ഗവേഷകൻ സംസാരിക്കുകയും, അവയെ വിമർശനാത്മകമായി പരിശോധിക്കുകയും ചെയ്തു.

കണ്ടെത്തലുകൾ : മിഷന്റെ മുഖ്യ അഭിലാഷം ആരോഗ്യ മേഖലയിലെ സാമൂഹ്യനീതിയിലധിഷ്ഠിതമായ തുല്യത എന്നതായിരുന്നെങ്കിലും, ജാതീയമായി നിലനിൽക്കുന്ന അനീതികളെ അഭിസംബോധന ചെയ്യുവാൻ കുടുംബാരോഗ്യ നയമോ, ആരോഗ്യവകുപ്പിലെ മുതിർന്ന ഉദ്യോഗസ്ഥരുടെ പ്രാഥമികാരോഗ്യ സേവനങ്ങളോടുള്ള കാഴ്ചപ്പാടുകളോ കണക്കിലെടുക്കാൻ മുതിർന്നിട്ടില്ല എന്ന് അനുമാനിക്കാം. ജാത്യധിഷ്ഠിതമായ പ്രശ്നത്തെ മൂന്ന് വിധത്തിൽ ഈ മിഷന്റെ നയപരിപാടിയിൽ അവഗണിച്ചതായി കാണപ്പെട്ടു. ഒന്ന്, അദൃശ്യമെങ്കിലും ജാതിബദ്ധമായി നിലനിൽക്കുന്ന വിവേചനം ദളിത് വിഭാഗത്തെ ബാധിക്കുന്നതിനെ (അ)ബോധപൂർവ്വം പരിഗണിച്ചില്ല. കൂടാതെ ജാതിപരിഗണനകളില്ലാതെ ദളിത് വിഭാഗങ്ങളെ കേവലം പ്രാദേശികസമൂഹങ്ങളായി പരിഗണിച്ച് അവരെ അദൃശ്യമാക്കി നിർത്തുന്ന രീതി. രണ്ടാമതായി ജാതി അധിഷ്ഠിതമായ പരിഗണനകൾ സംസ്ഥാനത്തെ പ്രാഥമികാരോഗ്യപരിപാലനത്തിന് വിഘാതമായി കാണുന്ന നിലപാട്. അവസാനമായി, സാമൂഹിക ആരോഗ്യ ഘടകങ്ങളുടെ അരാഷ്ട്രീയവൽക്കരണവും അവയിലൂടെ ജാതി ഒരു സാമൂഹിക ആരോഗ്യ

നിർണ്ണയ ഘടകമല്ലാതായി പരിഗണിക്കപ്പെടുന്ന സാഹചര്യവും മേൽപ്പറഞ്ഞ നയത്തിലും ആരോഗ്യ പ്രവർത്തകരുടെ കാഴ്ചപ്പാടിലും നിലനിൽക്കുന്നതായി കാണപ്പെട്ടു.

ഉപസംഹാരം: ആർദ്രം മിഷനും അതിലൂടെ ഉയർന്നു വന്നിരിക്കുന്ന സാമൂഹിക നീതിയെ പറ്റിയുള്ള ചർച്ചകളും പൂർണ്ണ അർത്ഥത്തിൽ വിജയിക്കണമെങ്കിൽ കേരളത്തിന്റെ പൊതുജനാരോഗ്യശാക്തീകരണനയത്തിൽ പ്രച്ഛന്നമായ നിൽക്കുന്ന ജാതിരാഹിത്യം പ്രശ്നവൽക്കരിക്കേണ്ടതുണ്ട്. ജാതിവിഭജനത്താൽ സംഭവിക്കുന്ന അവസരതുല്ല്യതാരാഹിത്യം പരിഗണനാ വിഷയമാവേണ്ടതുണ്ട്. അതാവും ഒരുപക്ഷെ സാമൂഹ്യനീതിയും അവസരതുല്ല്യതയും പൂർണ്ണമായും നടപ്പിലാവാൻ ഉതകുന്ന ഘടകം.
