# Supplementary material for: Correction: Leaving no one behind on the road to Universal Health Coverage: The Kerala story
Source: Int J Equity Health. 2024 Jul 9;23:137. doi: 10.1186/s12939-024-02195-3 (PMC11232204; doi:10.1186/s12939-024-02195-3)
Supplement: Supplementary file 1 — Supplementary Material 1. [file 12939_2024_2195_MOESM1_ESM.zip › 12939-2023-1981-9 Nayar.pdf]

Equity issues in gender-affirming medical care in Kerala: a reflective commentary
K. Rajasekharan Nayar & S. Vinu

ട്രാൻസ്ജെൻഡർ വ്യക്തിയുടെ സ്വന്തം ലിംഗ വ്യക്തിത്വത്തെ സംബന്ധിച്ച പരിവർത്തനവുമായി ബന്ധപ്പെട്ട മെഡിക്കൽ സേവനങ്ങൾ നൽകുന്നതാണ് ലിംഗഭേദം സ്ഥിരീകരിക്കുന്ന മെഡിക്കൽ പരിചരണം. ലിംഗമാറ്റം എന്നത് സാമൂഹിക പിന്തുണ മാത്രമല്ല മാനസികവും വൈദ്യപരവുമായ പിന്തുണയും ആവശ്യമുള്ള ഒരു പ്രക്രിയയാണ്. ഈ പ്രബന്ധം കേരളത്തിൽ ലിംഗഭേദം സ്ഥിരീകരിക്കുന്ന മെഡിക്കൽ പരിചരണത്തിന്റെ പശ്ചാത്തലത്തിൽ ട്രാൻസ്ജെൻഡർ വ്യക്തികൾ (ടിജി) നേരിടുന്ന വെല്ലുവിളികൾ രേഖപ്പെടുത്താൻ ശ്രമിക്കുന്നു. അത്തരം പ്രക്രിയയുടെ മുൻഗണന വ്യത്യസ്തമായതിനാൽ പരിവർത്തന പ്രക്രിയ വളരെ സങ്കീർണ്ണമാണ്. ചില ട്രാൻസ്ജെൻഡർ വ്യക്തികൾ അവരുടെ ലിംഗഭേദം അവരുടെ ലിംഗവ്യത്യാസവുമായി വിന്യസിക്കാൻ സാമൂഹിക പരിവർത്തനത്തിനമോ /അല്ലെങ്കിൽ മെഡിക്കൽ പരിവർത്തനത്തിനോ മുൻഗണന നൽകും, എന്നാൽ മറ്റുചിലർ പരമ്പരാഗത ലിംഗ ബൈനറിക്ക് പുറത്തു ഒരു ലിംഗ പദപ്രയോഗമോ ഐഡന്റിറ്റിയോ തിരഞ്ഞെടുക്കാൻ തീരുമാനിക്കും. കേരളത്തിൽ, സജീവമായ നയവും നല്ല നിയമപരമായ പിന്തുണയും ഉണ്ടായിരുന്നിട്ടും, ട്രാൻസ്ജെൻഡർ വ്യക്തികൾ ലിംഗഭേദം സ്ഥിരീകരിക്കുന്ന മെഡിക്കൽ പരിചരണത്തിൽ നിരവധി വെല്ലുവിളികൾ നേരിടുന്നു, അതിൽ കുടുംബ പിന്തുണയുടെ അഭാവവും ആരോഗ്യ സേവനങ്ങൾ ഉൾപ്പെടെ നിരവധി സാമൂഹിക പിന്തുണാ സ്ഥാപനങ്ങളുമായി ബന്ധപ്പെട്ട് ഇക്വിറ്റിയുമായി ബന്ധപ്പെട്ട പ്രശ്നങ്ങളും ഉൾപ്പെടുന്നു. മെഡിക്കൽ പാഠ്യപദ്ധതിയിലെ മാറ്റങ്ങൾ, കൂടുതൽ സജീവമായ സാമൂഹിക പിന്തുണ, ആരോഗ്യ പ്രവർത്തകർ ഉൾപ്പെടെയുള്ള സമൂഹത്തിന്റെ ബോധവൽക്കരണം എന്നിങ്ങനെ സാധ്യമായ ചില ഇടപെടലുകൾ നിർദ്ദേശിക്കപ്പെടുന്നു.
