# Supplementary material for: Correction: Leaving no one behind on the road to Universal Health Coverage: The Kerala story
Source: Int J Equity Health. 2024 Jul 9;23:137. doi: 10.1186/s12939-024-02195-3 (PMC11232204; doi:10.1186/s12939-024-02195-3)
Supplement: Supplementary file 1 — Supplementary Material 1. [file 12939_2024_2195_MOESM1_ESM.zip › 12939-2023-1991-7 Chandru.pdf]

Factors affecting ability of TB patients to follow treatment guidelines " applying a capability approach"
B Aravind Chandru and Ravi Prasad Varma

ക്ഷയരോഗികളുടെ ചികിത്സാ മാർഗ്ഗനിർദ്ദേശങ്ങൾ പാലിക്കാനുള്ള കഴിവിനെ ബാധിക്കുന്ന ഘടകങ്ങൾ - ഒരു കേപ്പബിലിറ്റി (കെൽപ് ആസ്പദം ആക്കിയുള്ള) സമീപനം

ബി അരവിന്ദ് ചന്ദ്രു & രവി പ്രസാദ് വർമ്മ
അച്യുത മേനോൻ സെന്റർ ഫോർ ഹെൽത്ത് സയൻസ് സ്റ്റഡീസ്, ശ്രീ ചിത്ര തിരുനാൾ ഇൻസ്റ്റിറ്റ്യൂട്ട് ഫോർ മെഡിക്കൽ സയൻസസ് ആൻഡ് ടെക്നോളജി, ട്രിവാൻഡ്രം, മെഡിക്കൽ കോളേജ് പോസ്റ്റ് ഓഫീസ്, തിരുവനന്തപുരം, കേരളം, ഇന്ത്യ.

ആമുഖം: ക്ഷയരോഗ ചികിൽസ എന്ന സങ്കീർണ്ണമായ പ്രക്രിയയിൽ നിശ്ചിത സമയങ്ങളിലും അളവുകളിലും ദിവസേന വിവിധ തരം മരുന്നുകൾ കഴിക്കുന്നതും ആനുകാലിക തുടർനടപടികളും തുടർ പരിശോധനകൾ നടത്തുന്നതും എല്ലാ ക്ഷയ രോഗം ബാധിതരിൽ ഏകീകൃതമായിരിക്കില്ല, ചിലർക്ക് മറ്റുള്ളവരേക്കാൾ മികച്ച രീതിയിൽ കൃത്യമായ ചികിൽസ നിർവഹിക്കാൻ സാധിച്ചേക്കാം. ഈ സാഹചര്യത്തിൽ, കേരളത്തിലെ തിരുവനന്തപുരം ജില്ലയിലെ ക്ഷയരോഗ ബാധിതരുടെ ഇടയിൽ ചികിത്സാ മാർഗ്ഗനിർദ്ദേശങ്ങൾ പാലിക്കാനുള്ള കഴിവ് കണ്ടെത്തുന്നതിനായി ഞങ്ങൾ ഒരു പഠനം നടത്തി.

രീതിശാസ്ത്രം: മിശ്രിതമായ രീതികൾ ഉൾച്ചേർത്ത ഒരു മാതൃകയാണ് ഈ പഠനം ഉപയോഗിച്ചത്. കേരളത്തിലെ തിരുവനന്തപുരത്ത് 18 വയസോ അതിൽ കൂടുതലോ പ്രായമുള്ള സാധാരണ മരുന്നുകളോട് പ്രതികരിക്കുന്ന ശ്വാസകോശ ടിബിയുള്ള 135 രോഗികളിൽ നിന്ന് ഞങ്ങൾ ക്രോസ്സ്-സെക്ഷണൽ (ഏകദേശം ഒരേ സമയം വിവരശേഖരണം നടത്തുന്ന പ്രക്രിയ) വിവരങ്ങൾ ശേഖരിച്ചു, എല്ലാ ചികിത്സാ മാർഗ്ഗനിർദ്ദേശങ്ങളും പാലിക്കുന്ന രോഗികളുടെ അനുപാതം കണ്ടെത്തുന്നതിന് വേണ്ടി ഒരു ഘടനാപരമായ ചോദ്യാവലി ഉപയോഗിച്ചു. സർവേ സാമ്പിളിൽ നിന്ന് ഞങ്ങൾ എട്ട് വിശദമായ അഭിമുഖങ്ങളും (നാല് പുരുഷന്മാരും നാല് സ്ത്രീകളും) നടത്തി. ചികിത്സാ മാർഗ്ഗനിർദ്ദേശങ്ങളുമായി സംബന്ധിച്ച് പഠനപങ്കാളികൾ എടുത്ത് തീരുമാനങ്ങൾ /നിലപാടുകൾ, അവയുടെ കാരണങ്ങൾ എന്നിവയെക്കുറിച്ച് വിപുലമായ ഉൾക്കാഴ്ചകൾ ലഭിക്കുന്നതിന് ആഴത്തിലുള്ള വിശദമായ അഭിമുഖങ്ങൾ ഇൻഡക്റ്റീവ് (നിർദ്ദിഷ്ട നിരീക്ഷണങ്ങളിൽ നിന്ന് വിശാലമായ സാമാന്യവൽക്കരണങ്ങളിലേക്ക് നീങ്ങുന്ന) രീതിയിൽ വിശകലനം ചെയ്തു. പങ്കെടുത്ത എല്ലാവരിൽ നിന്നും രേഖാമൂലമുള്ള സമ്മതം വാങ്ങുകയും ആവശ്യമായ സംസ്ഥാനക്ഷയ രോഗ പ്രോഗ്രാമമിന്റെയും മറ്റ്

നൈതീക നിയമാവശ്യകമായ അനുമതികളും എടുത്ത ശേഷമാണ് പഠനം നടപ്പിലാക്കിയത് .

കണ്ടെത്തലുകൾ: 105 പുരുഷന്മാരും 30 സ്ത്രീകളും ആണ് പഠനത്തിൽ പങ്കെടുത്തത്, ഇതിൽ 80 പേർ പ്രതിദിനം തടസ്സങ്ങൾകൂടാതെ മരുന്ന് കഴിക്കുന്നതായി റിപ്പോർട്ട് ചെയ്തു (59.3%, 95% CI 50.8-67.2%). മൊത്തത്തിൽ, 38 (28.2%, 95% CI 21.3%-36.3%) വ്യക്തികൾക്കും പഠനത്തിന് വിധേയമാക്കിയ ഏഴ് വശങ്ങൾ അടങ്ങിയ ചികിത്സാ മാർഗ്ഗനിർദ്ദേശങ്ങളും പിന്തുടരാൻ കഴിഞ്ഞിരുന്നു. വിപുലീകൃത/കൂട്ടുകുടുംബത്തിൽ താമസിക്കുന്നതും ((AOR) 2.6, 95% CI 1.1-6.0), ഏകദേശം 13,500 രൂപയിൽ കൂടുതൽ പ്രതിമാസ ഗാർഹിക ചിലവുകൾ ഉള്ളവരും (AOR 2.9, 95% CI 1.3–6.7) കൂടാതെ കാലതാമസം ഇല്ലാതെ പ്രാരംഭ പരിചരണം സ്വീകരിക്കുന്നതും (AOR 3.2, 95% CI 1.2–8.7) ചികിത്സാ മാർഗ്ഗനിർദ്ദേശങ്ങളുടെ എല്ലാ വശങ്ങളും പാലിക്കുന്നതുമായി ബന്ധം ഉള്ളതായി കാണപ്പെട്ടു . ശാരീരികാനുഭവങ്ങൾ, ധാർമ്മികകടമയുമായി ബന്ധപ്പെട്ട ധാരണകൾ, ക്ഷയരോഗത്തിന്റെ സാമൂഹിക നിർമ്മിതമായ കാഴ്ചപ്പാടുകൾ, നിയന്ത്രണ പദ്ധതി സംബന്ധമായ ഘടകങ്ങൾ, ലഹരിവസ്തുക്കളുടെ ഉപയോഗം എന്നിവ ചികിത്സയുമായി ബന്ധപ്പെട്ട പെരുമാറ്റങ്ങളെ സ്വാധീനിച്ചതായി വിശദമായ അഭിമുഖങ്ങൾ വെളിപ്പെടുത്തി. നിയന്ത്രണ പദ്ധതിയുടെ ഊന്നൽ വ്യക്തിപരമായ പ്രവർത്തനശേഷികളിൽ ആയിരുന്നെങ്കിലും ഇവയ്ക്കുള്ള കഴിവും, അനുകൂല സന്ദർഭങ്ങളും സ്വാധീനിക്കുന്നത് ടിബിയുമായി ബന്ധപ്പെട്ട അപമാനത്തിന്റെ അനുഭവം, ജൻഡർ (സാമൂഹിക നിർമ്മിതമായ സ്ത്രീപുരുഷന്മാരുടെ ലിംഗംഭേദം), ദാരിദ്ര്യം എന്നീ സാമൂഹികമായി ഭാവങ്ങൾ ആയിരുന്നു.

ഉപസംഹാരം : ജൈവശാസ്ത്ര ചികിത്സാരീതിപരമായും, സാമൂഹികമായും ഉള്ള പല പ്രശ്നങ്ങളുടെ ഒരു സിൻഡമിക്കിന്റെ നടുവിലാണ് ക്ഷയ രോഗികൾ ജീവിക്കുന്നത്. ഇത്തരം ക്ഷയരോഗികളുടെ ചികിത്സാ മാർഗ്ഗനിർദ്ദേശങ്ങൾ പാലിക്കാനുള്ള കഴിവുകളെയും അനുകൂല സന്ദർഭങ്ങളെയും ഈ പ്രശ്നങ്ങൾ സ്വാധീനിക്കുന്നു. ക്ഷയരോഗം നിയന്ത്രിക്കുന്നതിനുള്ള ഇടപെടലുകൾ വ്യക്തിഗത ഏജൻസിയിലും സാമൂഹിക സാമ്പത്തിക ഘടകങ്ങളിലും ഒരുപോലെ ശ്രദ്ധ കേന്ദ്രീകരിക്കേണ്ടത് അനിവാര്യമാണ്.
