# Supplementary material for: Correction: Leaving no one behind on the road to Universal Health Coverage: The Kerala story
Source: Int J Equity Health. 2024 Jul 9;23:137. doi: 10.1186/s12939-024-02195-3 (PMC11232204; doi:10.1186/s12939-024-02195-3)
Supplement: Supplementary file 1 — Supplementary Material 1. [file 12939_2024_2195_MOESM1_ESM.zip › 12939-2023-2005-2 Sharma.pdf]

Assessing inequalities in publicly funded health insurance scheme coverage and out-of-pocket expenditure for hospitalization: findings from a household survey in Kerala

Santosh Kumar Sharma, Jaison Joseph, Hari Sankar D, Devaki Nambiar

പൊതുമേഖല ആരോഗ്യ ഇൻഷുറൻസ് പരിരക്ഷ പദ്ധതിയിലെ അസമത്വങ്ങളുടെയും ചികിത്സാ ചെലവുകളുടെയും പഠനം: കേരളത്തിലെ ഒരു ഗാർഹിക സർവേയിൽ നിന്നുള്ള കണ്ടെത്തലുകൾ

സന്തോഷ് കുമാർ ശർമ്മ, ജെയ്സൺ ജോസഫ്, ഹരി ശങ്കർ ഡി, ദേവകി നമ്പ്യാർ

ആമുഖം:സാർവത്രിക ആരോഗ്യ പരിരക്ഷയുടെ ഒരു പ്രധാന ഘടകവും എല്ലാവർക്കും ആരോഗ്യം എന്ന ലക്ഷ്യം കൈവരിക്കുന്നതിനുമായുള്ള ഒരു മാർഗമാണ് ജനങ്ങൾക്കു ആരോഗ്യ പരിരക്ഷ ഉറപ്പാക്കുക എന്നത്. ചികിത്സക്കായി ഉണ്ടാകുന്ന സാമ്പത്തിക ആഘാതങ്ങളിൽ നിന്ന് പരിരക്ഷ ലഭ്യമാക്കുന്നതിനും, പരിചരണം തേടുന്നതിനായി ഉണ്ടാകുന്ന അധിക ചിലവ് കുറയ്ക്കുന്നതിനുമുള്ള മാർഗങ്ങളിലൊന്നായി സർക്കാർ നടപ്പിലാക്കുന്ന ആരോഗ്യ ഇൻഷുറൻസ് പദ്ധതികളെ കണക്കാക്കപ്പെടുന്നു. ദക്ഷിണേന്ത്യൻ സംസ്ഥാനമായ കേരളത്തിന് സർക്കാർ ആരോഗ്യ ഇൻഷുറൻസ് പദ്ധതികൾ നടപ്പാക്കുന്നതിൽ ഒരു ദശാബ്ദക്കാലത്തെ പരിചയമുണ്ട്. നാളിതുവരെ, ഈ പദ്ധതികളുടെ സേവന വ്യാപ്തിയും, ടി പദ്ധതികൾ വഴി പരിചരണം തേടിയവർക്ക് ലഭ്യമായ ആനുകൂല്യങ്ങളെയും, സാമ്പത്തിക പരിരക്ഷയെയും പറ്റി വളരെ കുറച്ചു പഠനങ്ങൾ മാത്രമേ കേരളത്തിൽ നടന്നിട്ടുള്ളൂ. ഈ വിടവ് നികത്തുക എന്ന ലക്ഷ്യത്തോടെ, സർക്കാർ ഇൻഷുറൻസ് പരിരക്ഷയിലെ വ്യാപ്തിയും, അസമത്വവും, ദാതാക്കളുടെ തിരഞ്ഞെടുപ്പ്, ഇൻഷുർ ചെയ്തവരും ഇൻഷുറൻസ് ഇല്ലാത്തവരുമായ വ്യക്തികൾക്കിടയിൽ കേരളത്തിൽ കിടത്തിചികിത്സക്കാവശ്യമായ ശരാശരി ചെലവ് എന്നിവയും ഞങ്ങളുടെ ഈ പഠനത്തിലൂടെ പരിശോധിച്ചു.

രീതിശാസ്ത്രം 2019 ജൂലൈ മുതൽ ഒക്ടോബർ വരെയുള്ള കാലയളവിൽ കേരളത്തിലെ നാല് ജില്ലകളിൽ ഗാർഹിക തലത്തിൽ സർവേ നടത്തി. മൾട്ടിസ്റ്റേജ് റാൻഡം സാമ്പിൾ ഉപയോഗിച്ചു എട്ട് പ്രാഥമിക ആരോഗ്യ പരിപാലന കേന്ദ്രങ്ങളുടെ സേവന പരിധിയിൽ പെടുന്ന 3234 വീടുകൾ നിന്നുള്ള 13,064 വ്യക്തികളിൽ നിന്ന് ഡാറ്റ ശേഖരിച്ചു. ശേഖരിച്ച വിവരങ്ങൾ വിശകലനം ചെയ്യുണത്തിനായി വിവരണാത്മക സ്ഥിതിവിവരക്കണക്കുകൾ, ബൈവാരിയേറ്റ്, മൾട്ടി വേരിയേറ്റ് അനാലിസിസ് എന്നിവ ഉപയോഗിച്ചു. അസമത്വത്തിന്റെ സ്ലോപ്പ് ഇൻഡക്സ്

(എസ്ഐഐ), ആപേക്ഷിക അളവ് - ആപേക്ഷിക കോൺസെൻട്രേഷൻ ഇൻഡക്സ് (ആർസിഐ) എന്നിവ ഉപയോഗിച്ച് ഞങ്ങൾ സാമൂഹിക സാമ്പത്തിക അസമത്വങ്ങൾ വിലയിരുത്തി.

കണ്ടെത്തലുകൾ :ഞങ്ങളുടെ പഠനത്തിൽ പങ്കെടുത്തവരിൽ ഗണ്യമായ ഒരു വിഭാഗം സർക്കാർ ആരോഗ്യ ഇൻഷുറൻസ് പദ്ധതിയിൽ (45.8%) ഉൾപ്പെട്ടവരായിരുന്നു/ അംഗങ്ങളാണ്. കുറഞ്ഞ വാർഷിക വരുമാനമുള്ള കുടുംബങ്ങൾക്കാണ് ഉയർന്ന വാർഷിക വരുമാനമുള്ള കുടുംബങ്ങളെക്കാൾ ആരോഗ്യ ഇൻഷുറൻസ് പരിരക്ഷ ലഭിക്കാൻ സാധ്യത. ആർസിഐ [-16.8% (95% CI: -25.3, -8.4)] എസ്ഐഐ [-21.5% (95% CI: -36.1, -7.0)] എന്നിവയുടെ നെഗറ്റീവ് കാന്തിമാനം ദരിദ്രർക്കിടയിൽ പിഎഫ്എച്ച്ഐഎസ് കവറേജിന്റെ ഉയർന്ന സാന്ദ്രത സൂചിപ്പിക്കുന്നു. സർക്കാർ ആരോഗ്യ ഇൻഷുറൻസ് പദ്ധതിയിൽ അംഗമായിട്ടുള്ളവർ സ്വകാര്യ ആശുപത്രി വഴി കിടത്തിച്ചികിത്സ തേടുന്നതിന് ശരാശരി 9000 രൂപ സ്വന്തം കയ്യിൽ നിന്നും ചിലവാക്കുന്ന. അതേസമയം ആരോഗ്യ ഇൻഷുറൻസ് പരിരക്ഷ ഇല്ലാത്തവർ സ്വകാര്യ ആശുപത്രികൾ വഴി കിടത്തിച്ചികിത്സ തേടുന്നതിന് 10500 രൂപ സ്വന്തം കയ്യിൽ നിന്ന് ചെലവാക്കുന്നു

ഉപസംഹാരം:പിഎഫ്എച്ച്ഐഎസ് ദരിദ്രരായ ജനസംഖ്യയെ ഉചിതമായി ലക്ഷ്യമിടുന്നതായി തോന്നുമെങ്കിലും, ഇൻഷ്വർ ചെയ്തവർക്കും ആശുപത്രിയിൽ അഡ്മിറ്റ് ആകുമ്പോൾ കയ്യിൽ നിന്നും തുക ചെലവാക്കേണ്ടി വരുന്നു, ഇൻഷുറൻസ് ഇല്ലാത്തവരിൽ, സാമ്പത്തികമായി മുന്നോക്കം ജനസംഖ്യാ ഉപഗ്രൂപ്പുകൾ ഏറ്റവും കൂടുതൽ തുക ചെലവഴിക്കുന്നു, ഇത് ആപേക്ഷികമായി പിന്നോക്കം നിൽക്കുന്നവർ പരിചരണം പൂർണ്ണമായും ഉപേക്ഷിക്കുകയാണോ അതോ ചികിത്സാ ചെലവ്കുറഞ്ഞ ഇടങ്ങളിൽ, അല്ലെങ്കിൽ സൗജന്യമായ സർക്കാർ സൗകര്യങ്ങളെ ഉപയോഗിച്ച് മാത്രമായോ പരിചരണം തേടുകയാണോ എന്ന ചോദ്യങ്ങൾ ഉയർത്തുന്നു. പി.എഫ്.എച്ച്.ഐ.എ സ് ഗുണഭോക്താക്കളിൽ സാമ്പത്തിക ഭാരം ഫലപ്രദമായി കുറയ്ക്കുന്നതിനുള്ള കൂടുതൽ നയപരമായ നടപടികൾ സംസ്ഥാനത്തെ യു.എച്ച്.സി പുരോഗതിക്ക് അത്യന്താപേക്ഷിതമാണ്
