# Supplementary material for: Correction: Leaving no one behind on the road to Universal Health Coverage: The Kerala story
Source: Int J Equity Health. 2024 Jul 9;23:137. doi: 10.1186/s12939-024-02195-3 (PMC11232204; doi:10.1186/s12939-024-02195-3)
Supplement: Supplementary file 1 — Supplementary Material 1. [file 12939_2024_2195_MOESM1_ESM.zip › 12939-2023-2090-3 Vincent.pdf]

What our children lost and gained at the time of school closure during the Covid-19 pandemic: a study on psychological distress, behavioural concerns and protective factors of resilience among preschool children in Kerala, India.

Jose Vincent, Resmi Madhusoodanan Santhakumari, Anjana Nalinakumari Kesavan Nair, Anisha Sharahudeen, Asvini K.P, Meenu Maheswari Suresh, Mathew J. Valamparampil, Gayathri A.V, Chintha Sujatha and Anish Thekkumkara Surendran

കോവിഡ് മഹാമാരിക്കാലത്ത് നമ്മുടെ കുട്ടികൾ നഷ്ടപ്പെടുത്തിയതും നേടിയതും : കേരളത്തിലെ മൂന്ന് മുതൽ അഞ്ച് വയസ്സ് വരെ ഉള്ള കുട്ടികളിലെ ദോഷകരമായ സ്വഭാവ സവിശേഷതകൾ, മാനസിക ബുദ്ധിമുട്ടികൾ, സംരക്ഷണഘടകങ്ങൾ എന്നിവയെ മഹാമാരിയുമായി ബന്ധപ്പെട്ട നിയന്ത്രണങ്ങൾ എങ്ങനെയാണ് ബാധിച്ചത്.

ജോസ് വിൻസെന്റ്, രശ്മി മധുസൂദനൻ ശാന്തകുമാരി, അഞ്ജന നളിനി കേശവൻ നായർ, അനീഷ ഷറഹുദ്ദീൻ, അശ്വിനി കെ.പി, മീനു മഹേശ്വരി സുരേഷ്, മാത്യു ജെ.വലംപറമ്പിൽ, ഗായത്രി എ.വി,ചിന്ത സുജാത.അനീഷ് തെക്കുംകര സുരേന്ദ്രൻ

ആമുഖം: സാമൂഹികമായ ഇടപടലുകൾ കുഞ്ഞുങ്ങളുടെ വൈകാരികവും സാമൂഹികവുമായ വളർച്ചക്ക് അത്യന്താപേക്ഷിതമാണ്. മഹാമാരിക്കാലത്ത് അതിനുള്ള സാധ്യതകൾ കുറഞ്ഞത്, പ്രത്യേകിച്ചും സ്കൂളുകൾ പോലെയുള്ള സ്ഥാപനങ്ങൾ നീണ്ടൊരു കാലം അടഞ്ഞ കിടന്നത് നമ്മുടെ കുഞ്ഞുങ്ങളെ ദോഷകരമായി ബാധിച്ചിരിക്കാൻ ഇടയുണ്ട്.
കുഞ്ഞുങ്ങളുടെ സാമൂഹികവും മാനസികവുമായ വളർച്ചക്ക് ഉതകുമായിരുന്ന- കുഞ്ഞുങ്ങൾ തമ്മിൽ ഉള്ള ഇടപെടലുകൾ, കുടുംബവുമായുള്ള ഇടപെടലുകൾ, സ്കൂളിൽ വച്ചുള്ള സാമൂഹിക ഇടപെടലുകൾ തുടങ്ങിയവ എത്ര മാത്രം ഈ മഹാമാരിക്കാലത്ത് നഷ്ടപ്പെട്ടു എന്ന കാര്യവും അത് എത്രമാത്രം കുഞ്ഞുങ്ങളുടെ മാനസികവും വൈകാരികവുമായ വളർച്ചയിൽ സ്വാധീനം ചെലുത്തി എന്നുള്ളതും പഠിക്കാനാണ് ഇവിടെ ഉദ്യമിച്ചിരിക്കുന്നത്.

രീതിശാസ്ത്രം :മൂന്ന് വയസ്സ് മുതൽ അഞ്ച് വയസ്സ് വരെ പ്രായമുള്ള 535 കുഞ്ഞുങ്ങളിൽ ഒരു സർവ്വേ രൂപത്തിലാണ് ഈ പഠനം നടത്തിയിട്ടുള്ളത്. കേരളത്തിലെ അഞ്ച് ജില്ലകളിൽ നിന്നുള്ള കുഞ്ഞുങ്ങൾ പഠനത്തിൽ പങ്കെടുത്തു. Devereux Early Childhood Assessment P2(DECA P2) എന്ന ചോദ്യാവലിയാണ് പഠനത്തിനായി ഉപയോഗിച്ചിട്ടുള്ളത്. കുഞ്ഞുങ്ങളിൽ മഹാമാരിക്കാലത്ത്

ഉണ്ടാകാനിടയുള്ള സ്വഭാവ സവിശേഷതകളുമായും മാനസിക വ്യാപാരങ്ങളായും, സാമൂഹിക ഇടപെടലുകളായും ബന്ധപ്പെട്ട അപകട സാധ്യതകളും, ഈ കുഞ്ഞുങ്ങൾ അതിനെതിരെ സ്വീകരിച്ചിട്ടുള്ള പ്രതിരോധ മാർഗ്ഗങ്ങളും മനസ്സിലാക്കാൻ ശ്രമിക്കുന്നതായിരുന്നു പഠനത്തിനുപയോഗിച്ച ചോദ്യാവലി.

കണ്ടെത്തലുകൾ :ബഹുഭൂരിപക്ഷം കുഞ്ഞുങ്ങൾക്കും സംരക്ഷണ ഘടകങ്ങൾ (protective factors) ഇപ്പോൾ ഉള്ളതിനേക്കാൾ കൂടുതൽ ആവശ്യമുണ്ട് എന്നാണ് പഠനം സൂചിപ്പിക്കുന്നത് (area of need എന്ന വിഭാഗത്തിൽപ്പെടുന്നു).
യഥാക്രമം 64.5%, 49%, 68.4% കുഞ്ഞുങ്ങൾക്ക് മറ്റുള്ളവരുമായി ബന്ധം സ്ഥാപിക്കുക (attachment/relationship), സ്വയം നിയന്ത്രിക്കുക(self-regulation, സ്വമേധയാ കാര്യങ്ങൾക്ക് തുടക്കം കുറിക്കുക (initiative) എന്നിവകളിൽ ആവശ്യമായത്ര പ്രാപ്തി ഇല്ലെന്ന് കണ്ടെത്തി.
മറ്റു ഘടകങ്ങളെ നിയന്ത്രിച്ചു കൊണ്ട് നടത്തിയ ലോജിസ്റ്റിക്ക് റിഗ്രഷൻ (Logistic Regression) മാതൃക പ്രകാരം പെൺകുട്ടികളെ അപേക്ഷിച്ച് ആൺകുട്ടികൾ', 'മറ്റു കുട്ടികളെ അപേക്ഷിച്ച് അമ്മമാരുടെ സമയം കുറച്ചു കിട്ടുന്ന കുഞ്ഞുങ്ങൾ', 'കുട്ടിയെ ശാന്തനാക്കാൻ ഇലക്ട്രോണിക് ഉപകരണങ്ങൾ ഉപയോഗപ്പെടുത്തുന്നു എന്ന വസ്തുത' എന്നിവ സ്വഭാവ സവിശേഷതകളെ ദോഷകരമായി (Area of need, Behavioural Concerns) ബാധിക്കുന്നതായി കണ്ടെത്തി.

ഉപസംഹാരം: പഠനവിധേയമായ നല്ലൊരു ശതമാനം കുട്ടികളിലും ദോഷകരമായ സ്വഭാവസവിശേഷതകൾ (behavioral concerns), വൈകാരികമായ പ്രതിരോധശേഷിക്ക് സഹായകമായ ഘടകങ്ങളുടെ (Protective factors) രാഹിത്യം എന്നിവ കാണപ്പെട്ടു. മഹാമാരിയുടെ ഭാഗമായി സാമൂഹിക ഇടപെടലിന് ലഭ്യമാകേണ്ട അവസരവും സമയവും നഷ്ടപ്പെട്ടത് ഇതിന് കാരണ മായിരിക്കാം. ഇലക്ട്രോണിക് ഉപകരണങ്ങളുമായി കൂടുതൽ സമയം ചിലവഴിക്കേണ്ടിവന്നത് മറ്റൊരു വെല്ലുവിളിയാണ്. കുട്ടികളുടെ പരിപാലനത്തിനായി , പ്രത്യേകിച്ച് അമ്മമാർക്ക് നീക്കിവയ്ക്കാൻ സാധിച്ച അധികസമയം മേൽപ്പറഞ്ഞ ദോഷകരമായ ഘടകങ്ങളുടെ സ്വാധീനങ്ങളെ ലഘൂകരിച്ചിരിക്കാനിടയുണ്ട് എന്നും പഠനം സൂചിപ്പിക്കുന്നു.
